# MHSA-EC: An Indoor Localization Algorithm Fusing the Multi-Head Self-Attention Mechanism and Effective CSI

**DOI:** 10.3390/e24050599

**Published:** 2022-04-25

**Authors:** Wen Liu, Mingjie Jia, Zhongliang Deng, Changyan Qin

**Affiliations:** School of Electronic Engineering, Beijing University of Posts and Telecommunications, Beijing 100876, China; jiamingjie@bupt.edu.cn (M.J.); dengzhl@bupt.edu.cn (Z.D.); qin_changyan@bupt.edu.cn (C.Q.)

**Keywords:** channel state information, multi-head self-attention, effective CSI, feature extraction, fingerprint localization

## Abstract

Channel state information (CSI) provides a fine-grained description of the signal propagation process, which has attracted extensive attention in the field of indoor positioning. The CSI signals collected by different fingerprint points have a high degree of discrimination due to the influence of multi-path effects. This multi-path effect is reflected in the correlation between subcarriers and antennas. However, in mining such correlations, previous methods are difficult to aggregate non-adjacent features, resulting in insufficient multi-path information extraction. In addition, the existence of the multi-path effect makes the relationship between the original CSI signal and the distance not obvious, and it is easy to cause mismatching of long-distance points. Therefore, this paper proposes an indoor localization algorithm that combines the multi-head self-attention mechanism and effective CSI (MHSA-EC). This algorithm is used to solve the problem where it is difficult for traditional algorithms to effectively aggregate long-distance CSI features and mismatches of long-distance points. This paper verifies the stability and accuracy of MHSA-EC positioning through a large number of experiments. The average positioning error of MHSA-EC is 0.71 m in the comprehensive office and 0.64 m in the laboratory.

## 1. Introduction

The positioning algorithm is the basis of Location-Based Service (LBS). People need location information in many scenes such as production and life, entertainment, and public services. Traditionally, LBS is limited to outdoor activities, but the expansion of building scale and the increase of people’s activities in indoor scenes have given indoor positioning a larger market. For indoor positioning, the outdoor positioning method cannot be copied, because the widely used Global Positioning System (GPS) positioning has very weak signals in the indoor environment. Common indoor wireless positioning technologies are as follows: based on Wi-Fi [1,2], Bluetooth [3,4], RFID [5], and Ultra-wideband (UWB) [6,7] positioning method.

Among them, the Wi-Fi-based positioning method has a wide range of applications, and the technology has developed from the initial positioning based on RSSI [8] to the positioning technology based on channel state information (CSI). Channel state information (CSI) refers to the known channel properties of a communication link. Compared with the traditional received signal strength (RSSI), CSI can describe the channel in more detail.

### 1.1. CSI-Based Localization

In the field of CSI-based indoor positioning, scholars initially believed that multi-path information was interference. Taking the relationship between distance and CSI signal as the starting point, by suppressing the multi-path effect, the relationship between CSI and distance was constructed after preprocessing. As for the positioning method of the ranging model, with the development of CSI in the field of positioning, more scholars have found that multi-path information is not all interference; it can identify fingerprint points with similar distances well and can greatly enhance the discrimination of signals between different fingerprint points. Therefore, fingerprint-based localization methods have gradually become popular.

The positioning system based on the ranging model realizes indoor positioning by constructing the relationship between CSI and distance. The main representative is the FILA system. The system uses CSI for the first time to establish a propagation model to achieve indoor positioning. The system “exploits the frequency diversity to compensate the small-scale fading effect” and finally models the relationship between the extracted ${CSI}_{eff}$ and the distance.

The fingerprint localization method is to match the test point to the nearest fingerprint point. CSI-MIMO [9] processes the features by making a difference in the amplitudes of adjacent subcarriers and then uses the KNN algorithm to achieve positioning. In addition to these methods of manually processing CSI features, many localization algorithms use machine learning models (such as Support Vector Machines (SVM), Random Forests (RF), Naive Bayes (NB), etc.) for feature extraction [10,11,12]. Zhou et al. [10] proposed an algorithm to remove outliers using clustering and used SVM regression to establish a nonlinear relationship between CSI fingerprints and locations. In the DeepFi algorithm [13], the fingerprint is replaced by the deep learning model weight, and then, the radial-based probabilistic method is used for position prediction. The positioning errors of the DeepFi algorithm in the open environment and complex environment are 0.94 m and 1.80 m, respectively. In the confi [14] algorithm, the CSI is restructured into a time-frequency tensor and utilizes a CNN network for feature extraction. The mean error of ConFi is about 1.36 m. Hsieh et al. [15] compared the four localization methods of MLP-RSS, MLP-CSI, CNN-RSS, and CNN-CSI. Compared with the Confi network, the sample here only uses the one-dimensional tensor of CSI (1D-CSI), and no timing information is introduced. However, the results indicate that the 1D-CNN using CSI information achieves excellent localization performance with much lower network complexity. The LC-DNN [16] algorithm validates the correlation between adjacent subcarriers and introduces the position-dependent local feature. On this basis, the algorithm adopts local connections with separate convolution kernels to extract position-dependent local features. The algorithm achieves a positioning accuracy of 0.78 m in indoor scenes.

### 1.2. Limitations

**Figure 1:**
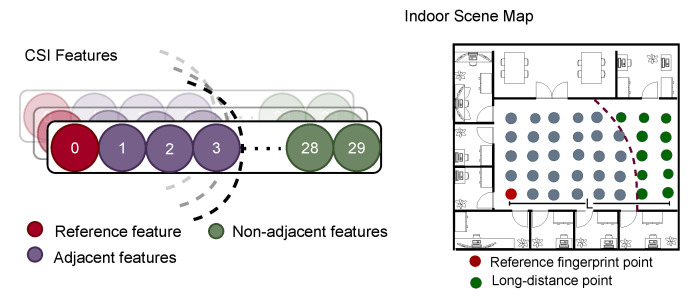
The non-adjacent features and the long-distance point.

The current mainstream CSI-based localization method is the fingerprint-based localization method using deep learning. There are still some limitations to this type of approach. We first address two problems: non-adjacent features aggregation and long-distance point mismatching. As shown in Figure 1, in the CSI features, we locate the features that are three dimensions away from the reference feature as adjacent features, and the features outside this are called non-adjacent features. As for the long-distance point, if the length of the indoor scene is L, we locate the point outside the reference point $\frac{3}{4}$L as the long-distance point. Here, we just want to emphasize the approximate distance of distant points, and different scenes have their own ratios.

CSI feature extraction based on convolutional neural network is still insufficient. In location feature extraction, multi-path information brings higher discrimination to points with similar distances. It is mentioned in [16,17] that the multi-path effect of a point is reflected in the correlation between the CSI subcarriers and the communication link. The current application uses the CNN network to aggregate the features of these two dimensions. However, CNN is limited by the small scale of the receptive field, and it is difficult for a single layer to aggregate all feature dimensions. In addition, some articles confirm that CNN is weaker than other methods in capturing non-adjacent feature dependencies. Section 1.3 goes into more detail.

Multi-path information brings higher discrimination to different points but also makes it hard to map the feature differences between fingerprint points to distances. In other words, it is hard to establish a clear mapping relationship with the distance for the raw CSI features that have not been processed. Therefore, when a mismatch occurs, such methods can match any fingerprint point in the absence of distance constraints. If the algorithm incorrectly matches the test point to the long-distance fingerprint point, the positioning accuracy will be greatly reduced.

### 1.3. Attention Mechanism

The change from MLP to CNN has gradually improved the CSI positioning performance. It can be seen that the network feature extraction ability directly affects the positioning performance. At present, the attention mechanism is widely used in neural network architecture. Transformer [18] proposed by Google has better modeling for time series, and the accuracy and running speed are greatly improved. In the follow-up development, the transformer model has been widely used in the fields of time prediction and feature mining. Botnet [19] is a simple yet powerful backbone that incorporates self-attention into a variety of computer vision tasks, replacing spatial convolution with global self-attention in the last three bottleneck blocks of ResNet. The model improves the baseline in instance segmentation and object detection while also reducing parameters. The ViT [20] divides the image into blocks, inputs the image features of different blocks into the transformer neural network, and achieves a good classification effect. This paper confirms that the reliance on CNNs is not necessary, and a pure transformer applied directly to sequences of image patches can perform very well in image classification tasks.

In particular, this article [21] mentioned that on the subject–verb agreement problem, the performance of the transformer is better than that of the CNN network model. From this experiment, it can be seen that the transformer is more advantageous in dealing with the long-distance dependence of features. Inspired by this article, we consider applying the attention mechanism of the transformer model to the indoor localization task of CSI. In CSI positioning, the correlation of subcarriers embodies multi-path information—a kind of information that is extremely related to location. This correlation is not limited to adjacent subcarriers but also exists between distant subcarriers. Therefore, in theory, the application of the attention mechanism in the transformer will have a positive effect on the CSI positioning task.

### 1.4. Contributions

**Figure 2:**
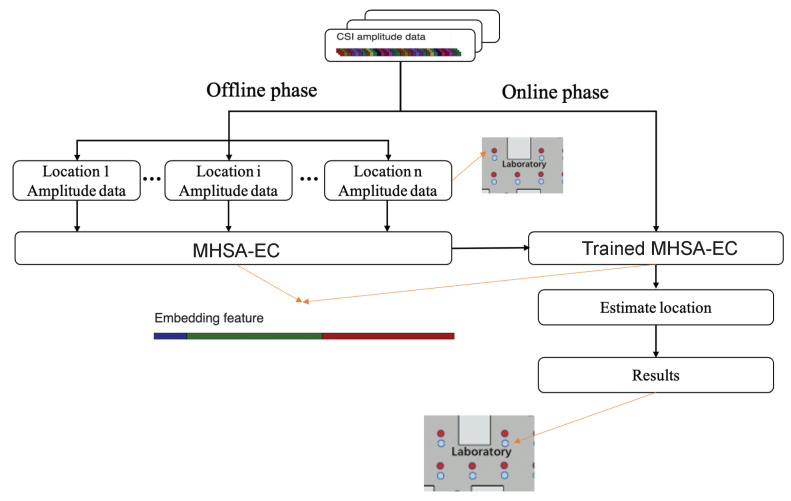
In the offline phase, the CSI data of each fingerprint point need to be collected for training the MHSA-EC model. In the online phase, the trained MHSA-EC model is deployed to predict the test point coordinates. The red point in the figure is the reference point, and the blue point is the test point.

In this paper, a multi-head self-attention mechanism and effective CSI fusion model (MHSA-EC) is constructed. MHSA-EC aims to solve the problem that the CNN model has insufficient ability to extract CSI features and the long-distance point mismatch problem that is easy to occur in the CSI fingerprint-based localization algorithm. MHSA-EC is used to simultaneously perform feature processing and distance information fusion on the CSI information collected from a single location point and construct a joint representation of multi-path information and distance information. The system block diagram is shown in Figure 2:

As an end-to-end network model, it is necessary to build a fingerprint database in the offline stage and train MHSA-EC to update the network weights. In the online stage, the trained MHSA-EC is deployed to meet the positioning requirements of online CSI data.

The MHSA-EC model proposes the following improvements and innovations for the limitations of CSI positioning:

1. The algorithm solves the problem of long-distance fingerprint point mismatch in the CSI-based fingerprint positioning algorithm. For the problem of mismatches that are prone to occur at farther distances, we introduce effective CSI as an input to the decision module. Since there is a nonlinear mapping relationship between effective CSI and distance, this signal is introduced to help the decision module to more effectively constrain the position output. The introduction of effective CSI can greatly increase the average positioning accuracy of the system.
2. The attention mechanism solves the problem of insufficient CSI feature extraction ability of the CNN network. The multi-path information contained in the subcarriers and arrays in the CSI signal increases the discrimination of CSI features. The CSI feature extraction method based on the CNN network is limited by the receptive field, and it is difficult to aggregate non-adjacent CSI features, resulting in the insufficient ability of the model to extract multi-path information. This paper improves the feature extraction capability of CSI signals by introducing the attention mechanism from a larger network receptive field and a better ability to aggregate non-adjacent features. The model’s ability to extract CSI features determines whether the model can correctly distinguish the CSI features of different fingerprint points. Therefore, the MHSA-EC model with better CSI feature extraction capability can theoretically improve the localization accuracy of the algorithm.
3. In addition, this paper also conducts extensive experiments to verify the localization performance of the network in two typical scenes. It also carries out ablation experiments to verify the effectiveness of the network module.

## 2. Preliminary

### 2.1. CSI

OFDM is a bandwidth-limited digital multi-carrier modulation method of wireless communication. Its modulation and demodulation are based on Inverse Fast Fourier Transform (IFFT) and Fast Fourier Transform (FFT), respectively. OFDM has become the most widely used multi-carrier modulation technique, whereas CSI is a sampling of the frequency response in an OFDM system. It can be obtained by inserting a reference signal at the transmitter and estimating the channel at the receiver:

Y=H·X+n

where *Y* represents the received signal, *n* is the additive white Gaussian noise, *X* is the transmitted signal, and *H* represents the channel state information. In practical applications, *H* can be estimated by the relationship between the received signal and the transmitted signal $\hat{H}$. The obtained $\hat{H}$ is the set of K subcarrier channel state information, which is denoted as $\hat{H}=\left[{\hat{h}}_{f_{0}},{\hat{h}}_{f_{1}},\ldots ,{\hat{h}}_{f_{K}}\right]$, where $f_{k}$ is the center frequency of the *k*th subcarrier. Each subcarrier channel state information ${\hat{h}}_{f_{k}}$ can be expressed as:

$${\hat{h}}_{f_{k}}=\left|{\hat{h}}_{f_{k}}\right|exp\left\{j∠{\hat{h}}_{f_{k}}\right\}$$

where $|{\hat{h}}_{f_{k}}|$ represents the amplitude value of the channel state information of the *k*th subcarrier, and $∠{\hat{h}}_{f_{k}}$ represents the phase angle of the subcarrier. In indoor positioning tasks based on CSI amplitude, the multi-path effect is ubiquitous, resulting in aliasing between subcarriers. Based on this problem, the concept of effective CSI is proposed:

$${CSI}_{eff}=\frac{1}{K}\sum_{\;k=1\;}^{K}\frac{f_{k}\;}{f_{0}}\times {|\hat{h}|}_{k},k\in \left(-15,15\right)$$

where $f_{0}$ is the center frequency, $f_{k}$ is the frequency of the *k*th subcarrier, and ${|\hat{h}|}_{k}$ is the amplitude of the CSI of the *k*th subcarrier. Effective CSI is used to exploit the frequency diversity to compensate for the small-scale fading effect. While reducing the fading effect, we explore the relationship between the distance between the transmitter and the receiver.

### 2.2. Self-Attention

In the field of natural language processing, transformers have excellent performance. The self-attention mechanism is an important method of the transformer model, which can better understand the semantic information by learning the word dependencies within the sentence. Its advantages lies in its low computational complexity, parallel computation, and better learning of long-distance dependencies. Its formula is as follows:

$$Attention\left(Q,K,V\right)=softmax\left(\frac{QK^{T}}{\sqrt{d_{k}}}\right)V$$

$$Q=W_{q}X$$

$$K=W_{k}X$$

$$V=W_{v}X$$

where *Q*, *K*, and *V* represent query, key, and value respectively, $d_{k}$ is the number of columns of *K*, X is the input and $W_{q}$, $W_{k}$, and $W_{v}$ are the weight matrices of *Q*, *K*, and *V* respectively.

## 3. Materials and Methods

### 3.1. CSI Tensor

In the process of fingerprint collection of CSI, for a fingerprint point *i*, the collected CSI amplitude can be expressed as:

$$A\left(i\right)=\left[\begin{array}{cccc} a_{i}^{11} & a_{i}^{12} & \cdots & a_{i}^{1N}\\ a_{i}^{21} & a_{i}^{22} & \cdots & a_{i}^{2N}\\ \vdots & \vdots & \ddots & \vdots \\ a_{i}^{M1} & a_{i}^{M2} & \cdots & a_{i}^{MN} \end{array}\right]$$

where $a_{i}^{mn}$ represents the CSI amplitude value of the *m*th link and the nth subcarrier at the *i* fingerprint point. The link here refers to the connection path between all the receiving ends and the transmitting ends of all the n-numbered subcarriers, so the number of M is equal to the product of the number of transmitter antennas and the number of receiver antennas. Considering the excellent performance of the self-attention mechanism in the sequence classification task, we vectorize the CSI signal at the fingerprint point into a 1*N*M three-dimensional tensor. At the same time, we adopt the normalization method to make the network converge faster during training. The maximum and minimum values of a CSI tensor will be recorded, and the CSI amplitude value of the *m*th row and *n*th column will be processed as follows:

$${\hat{a}}_{i}^{mn}=\frac{a_{i}^{mn}-Min\left(a_{i}^{mn}\right)}{Max\left(a_{i}^{mn}\right)-Min\left(a_{i}^{mn}\right)}$$

The normalized CSI tensor is as follows:

$$\hat{A}\left(i\right)=\left[\begin{array}{cccc} {\hat{a}}_{i}^{11} & {\hat{a}}_{i}^{12} & \cdots & {\hat{a}}_{i}^{1N}\\ {\hat{a}}_{i}^{21} & {\hat{a}}_{i}^{22} & \cdots & {\hat{a}}_{i}^{2N}\\ \vdots & \vdots & \ddots & \vdots \\ {\hat{a}}_{i}^{M1} & {\hat{a}}_{i}^{M2} & \cdots & {\hat{a}}_{i}^{MN} \end{array}\right]$$

The above formula is the CSI tensor in the subcarrier dimension. Considering the location information also covered in the antenna dimension, we transpose the raw CSI tensor and perform the same processing to obtain ${\hat{A}}^{T}$. The final network input is $\left[\hat{A}\;{\hat{A}}^{T}\right]$.

### 3.2. System Architecture

**Figure 3:**
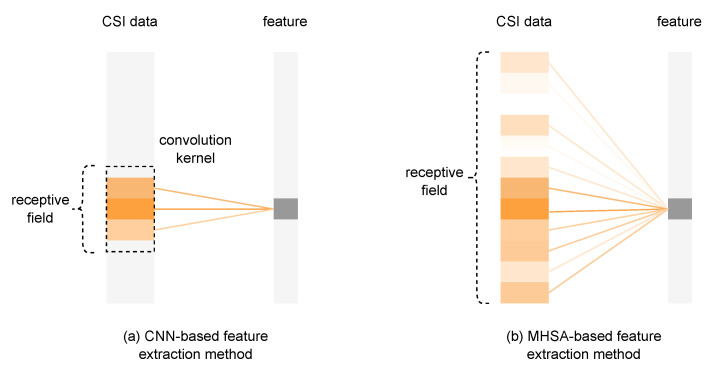
Convolutional neural network extraction method (**a**) compared with multi-head self-attention neural network extraction method (**b**). The shades of orange represent the weight of the feature.

There is still room for improvement in existing classical CSI-based localization models. Classic models still need improvement in CSI feature extraction. Classical networks such as Confi [14] and 1d-CNN [15] all use convolution operations as feature extraction methods. The problem of doing this can be represented in Figure 3:

Limited by the size of the receptive field, when the network aggregates features, it is difficult for CNN to aggregate all features in a single-layer network compared to the multi-head self-attention method (MHSA). However, there is a correlation between non-adjacent carriers and antennas in the CSI signal, and mining this correlation is very helpful for capturing multi-path information and improving positioning accuracy. Therefore, the feature extraction ability of the multi-head self-attention mechanism is better.

In addition, due to the fluctuation of the CSI signal, the positioning model may encounter the problem of mismatching distant points. The classical model lacks constraints for this problem, resulting in a high maximum positioning error. Introducing a kind of distance information to the model can effectively solve this problem.

**Figure 4:**
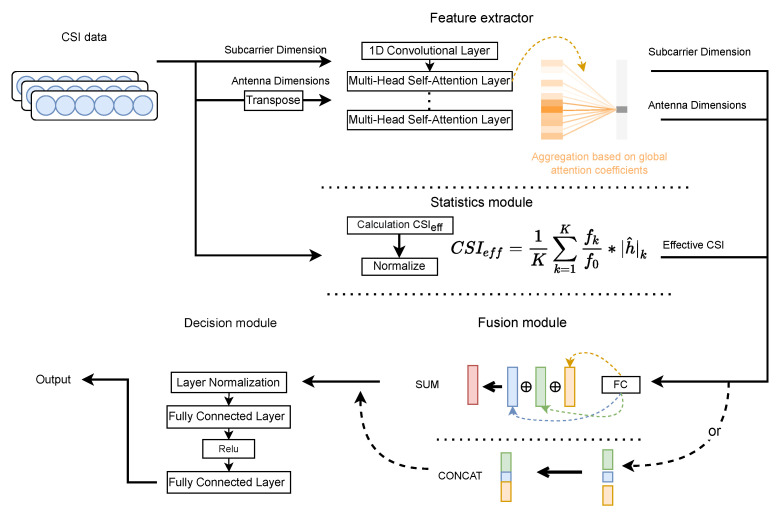
The processed CSI tensors are sent to the fusion module for data fusion after passing through the feature extractor and statistical module. The decision module fits the fused information to get the final position. The dotted line in the figure indicates that in the fusion algorithm, two methods of concatenation or summation can be selected. Section 3.2.3 will discuss this in more detail.

Based on the above discussion, we design the following network. MHSA-EC includes four modules: feature extraction, statistical module, fusion method, and fully connected decision. The structure is shown in Figure 4.

MHSA-EC first uses the multi-head self-attention mechanism to extract the key features of the subcarrier dimension and the antenna dimension, aiming at aggregating the relevant information between the carriers and between the antennas, and calculating the fingerprint characteristics of the current node. Effective CSI, a statistical vector that suppresses multi-path effects, is obtained through frequency diversity. Finally, the fusion module summarizes all the branch inputs to the decision module, and the decision module uses multiple fully connected layers and activation functions to perform fingerprint classification. The input in the network is the CSI amplitude, and the label is the position of the fingerprint point. The parameters are trained and optimized by gradient descent.

#### 3.2.1. Feature Extraction Module

**Figure 5:**
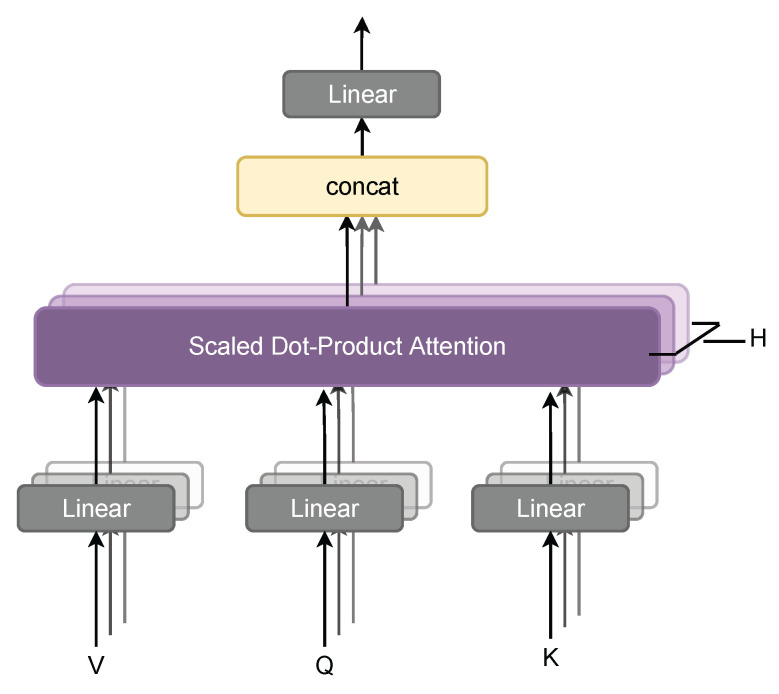
MHSA concatenates the results of each self-attention layer and then obtains the final result through a linear transformation.

Considering that the CSI dimension is less and it is difficult to mine potential features, the $\left[\hat{A},\;{\hat{A}}^{T}\right]$ is dimensionally expanded through the transformation of a one-dimensional CNN to obtain $\left[F_{carrier},F_{array}\right]$. For the problem of insufficient performance of the CNN model for long-distance subcarrier extraction of CSI, this paper proposes to use a multi-head self-attention mechanism to extract CSI features. Compared with the self-attention mechanism, the multi-head self-attention mechanism (MHSA) splices the outputs of multiple self-attentions to form multiple subspaces, ensuring that the model pays attention to different aspects of multi-path information. This extraction method also allows the model to advance to richer location features. The block diagram of the MHSA is shown in Figure 5:

In the MHSA layer, there are *H* self-attention layers, and the output of the self-attention layer whose index is i∈[1,H] is:

$$head_{carrier}^{i}=softmax\left[\frac{\left(W_{Q}^{i}F_{carrier}\right){\left(W_{K}^{i}F_{carrier}\right)}^{T}}{\sqrt{d_{F}}}\right]\left(W_{V}^{i}F_{carrier}\right)$$

$W_{Q}^{i}$, $W_{K}^{i}$, and $W_{V}^{i}$ correspond to the mapping weights of different linear layers. The function of softmax in the formula is as follows:

$$softmax\left(x_{i}\right)=\frac{exp(x_{i})}{\sum_{j}exp\left(x_{j}\right)}$$

where $x_{i}$ represents the *i*th eigenvalue. The feature extraction module contains *M* MHSA layers, and the output of the MHSA layer with index j∈[1,M] is:

$$mhsa_{carrier}^{j}=\left[head_{carrier}^{1};head_{carrier}^{2};\ldots ;head_{carrier}^{H}\right]W_{O}^{j}$$

$W_{O}^{j}$ is the linear layer weight passed after multiple heads are spliced. Multiple MHSA layers are concatenated to obtain the final output $mhsa_{carrier}$. The CSI of the antenna dimension will go through a similar extraction step and become $mhsa_{array}$. The output of the final feature module is $\left[mhsa_{carrier},mhsa_{array}\right]$.

#### 3.2.2. Effective CSI Statistics Module

Effective CSI is used to compensate for small-scale fading effects, which have a nonlinear relationship with physical distance. In FILA, the effective CSI is related to the distance as follows:

(1) $$d=\frac{1}{4\pi }{\left[{\left(\frac{c}{f_{0}*\left|CSI_{eff}\right|}\right)}^{2}*\sigma \right]}^{\frac{1}{n}}$$

where *c* is the wave velocity, σ is the environment factor, *n* is the path loss fading exponent, and $f_{0}$ is the central frequency. Based on this relationship, the introduced effective CSI can be input to the network as a kind of distance constraint information, thereby alleviating the mismatch of distant points. Therefore, in the statistics module, the effective CSI is calculated, and the formula is as follows:

$$CSI_{eff}=\frac{1}{K}\sum_{k=1}^{K}\frac{f_{k}}{f_{0}}*{\left|\hat{h}\right|}_{k},k\in \left(-15,15\right)$$

where *k* is the subcarrier index, generally IEEE 802. An 11n standard commercial wireless network card can collect 30 subcarrier numbers, where $f_{0}$ is the center frequency, and $f_{k}$ is the frequency of the *k*th subcarrier. $|\hat{h}{|}_{k}$ is the *k*th subcarrier CSI amplitude. Considering the variable offset that may exist in network training, layer normalization is performed on the $CSI_{eff}$ signal to obtain $\hat{CSI_{eff}}$.

#### 3.2.3. Fusion and Position Determination

This paper considers two ways for the fusion of $\hat{CSI_{eff}}$ and $[{mhsa}_{carrier}$, ${mhsa}_{array}]$: concatenation and summation. The concatenation method will connect each feature end to end:

$$feat_{merged}={mhsa}_{carrier}\left|\right|{mhsa}_{array}\left|\right|\hat{CSI_{eff}}$$

The fusion method does not require dimension size and requires fewer parameters than the summation method. However, the feature dimension input to the decision module is larger. The summation method requires a dimension alignment of each feature:

$$feat_{S}=W_{S}*{mhsa}_{carrier}+b_{S}$$

$$feat_{A}=W_{A}*mhsa_{array}+b_{A}$$

$$feat_{C}=W_{C}*\hat{\;CSI_{eff}}\;+b_{C}$$

$$feat_{merged}=feat_{S}+feat_{A}+feat_{C}$$

$W_{*}$ and $b_{*}$ are the weights and biases of the linear layer corresponding to different input signals. The summation method has a lower dimension input to the decision-making module, and it can fuse the effective CSI with each CSI feature, and the fusion level is deeper. The experimental section is devoted to further research on the two fusion methods.

The decision module firstly normalizes each dimension of the fusion feature to speed up the convergence of the network. After that, the feature is passed through multiple modules of fully connected layers and nonlinear layers to fit the position of the fingerprint points. For the final position output, the network uses a cross-entropy loss function to guide the network optimization; the formula is:

$$H_{y}\left(\hat{y}\right)=-\sum_{i}y_{i}*log\left({\hat{y}}_{i}\right)$$

where $\hat{y}$ is the predicted probability distribution and *y* is the true probability distribution. By calculating the loss of predicted results and actual results, the optimization direction of the model is guided.

### 3.3. Optimization

In order to reduce the tedious manual parameter tuning, we choose the Hyperopt tool for automatic hyperparameter selection. Hyperopt uses a form of Bayesian optimization for parameter tuning that allows the user to obtain the best parameters for a given model. Therefore, we define the search space of parameters, where the batch-size search space is {16, 32, 64, 128}, nhead is {2, 3, 4, 5, 6}, and lr is {0.001, 0.0025, 0.0005, 0.00075}. Then, we use Hyperopt for Bayesian optimization to output an optimal positioning network.

## 4. Experimental Verification

### 4.1. Experimental Parameters and Scenes

The positioning stability and accuracy of MHSA-EC are tested in two typical indoor scenes: comprehensive office and laboratory. The comprehensive office scene is larger and has more points, which is conducive to the stability of the test model and the maximum positioning error. In the laboratory, the overall area is small but contains rich multi-path information, so the positioning results here can reflect the model’s ability to extract features. On the acquisition device, both the transmitter and receiver are mobile devices equipped with an Intel 5300 NIC Terminal. Each terminal uses the Ubuntu 16.04 system. At the receiver, fine-grained CSI data are parsed by modifying the driver. The received packets include time stamp, RSSI, number of antennas, noise, CSI, etc.

#### 4.1.1. Experimental Parameters

The data set size is 232,000, of which 70% is the training set, 20% is the test set, and 10% is the validation set. There are specific parameters in Table 1: batch-size = 16, Learning-rate = 0.0005, the number of heads in the MHSA layer(nhead) = 5. The loss function is cross-entropy loss, and the activation function is Relu. In the training phase, we use the Glorot initializer to initialize the network. We use Adam as the gradient decay algorithm, and we also use the early stopping strategy; the initial best loss = 1 × $10^{8}$.

#### 4.1.2. Laboratory

**Figure 6:**
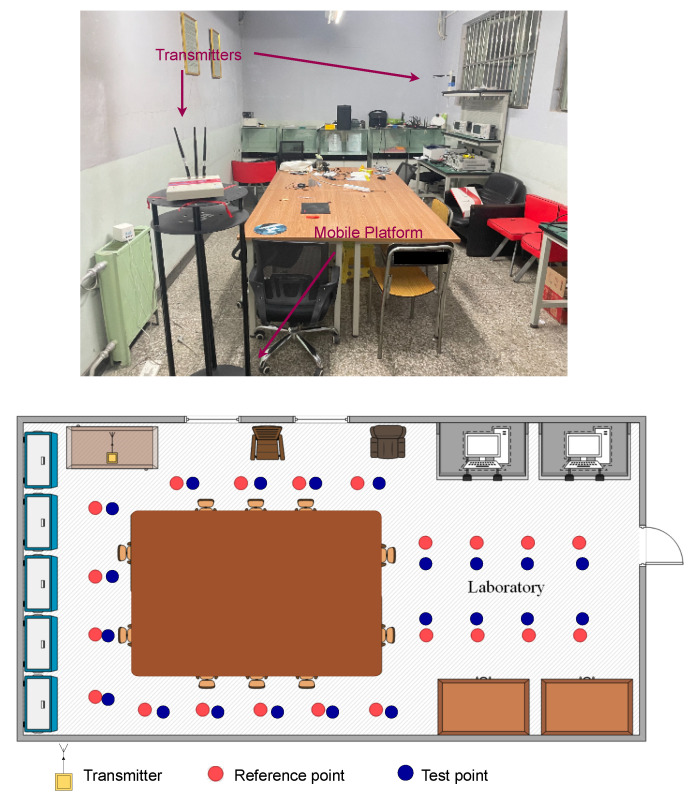
Layout of the laboratory.

As shown in Figure 6, We choose a standard laboratory with an area of 20 m${}^{2}$. The layout is shown in Figure 6. Its center is occupied by a large table with only a small number of personnel and some experimental equipment in the room. This is a typical indoor scene that contains rich multi-path information, which is very suitable for verifying the ability of our model to process multi-path information. The scene contains 20 reference points (red) and 1 transmitter (yellow). The distance point between two adjacent references is 0.5 m.

#### 4.1.3. Comprehensive Office

**Figure 7:**
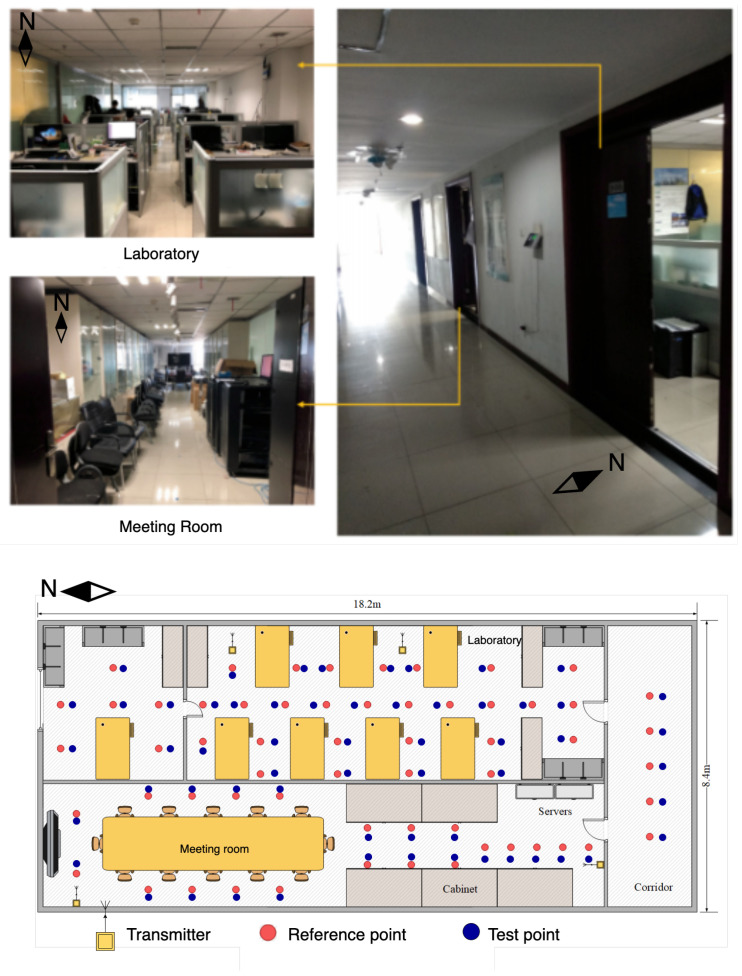
Layout of the comprehensive office.

As shown in Figure 7, this is a comprehensive office environment, including office areas, meeting rooms, and hallways. The total area is 152.9 m${}^{2}$; the conference room is 16.4 × 4.4 m${}^{2}$; the meeting room is 16.4 × 4 m${}^{2}$; and the corridor is 8.4 × 1.8 m${}^{2}$. The office area contains many desks and computer equipment. The office area and the conference room are separated by a glass wall. The entire scene contains 59 reference points and four transmitters. The distance between the two reference points is 1.2 m.

### 4.2. Model Performance Evaluation

#### 4.2.1. Multi-Head Self-Attention Layers Experiment

We determine the number of layers of the multi-head self-attention (MHSA) mechanism by comparing the experimental methods. Considering that the amount of data in the antenna branch and the subcarrier branch are the same, we keep the same number of layers on the two branches. The experiments were carried out in the comprehensive office scene. The results are shown in Table 2.

We investigate from the two experimental indicators, the mean absolute error (MAE) and the standard deviation (STD), and it can be seen that the accuracy and stability of the positioning increase significantly with the increase of the number of layers. After continuing to the fourth layer, the increase in the number of layers is not obvious. Based on the comprehensive consideration of model complexity and positioning time, we set the number of multi-head attention layers to three layers.

#### 4.2.2. Fusion Method Experiment

After fixing the network parameters, we study the fusion method of the effective CSI information and features. The experimental scene is also in the comprehensive office. We compared the fusion methods of concatenation and summation. The results are as follows: as can be seen from Table 3, compared with the concatenation method, the summation method has more advantages in terms of average positioning accuracy and model parameters, which is also due to the deeper fusion level of the summation method.

#### 4.2.3. Positioning Performance Evaluation

**Figure 8:**
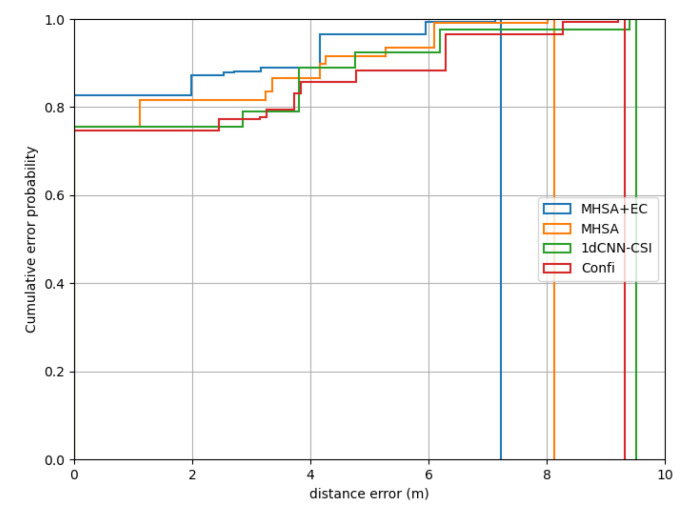
Performance on the laboratory.

We compare MHSA-EC with MHSA without effective CSI to show the $CSI_{eff}$ role in localization. At the same time, we also compared other existing methods in two scenes, including the 1dCNN-CSI algorithm in [15] and the mainstream Confi [14] system. The CSI magnitude signal is used by both systems and the MHSA-EC algorithm. The difference is that the 1dCNN-CSI method uses a one-dimensional CSI tensor such as MHSA-EC, while the Confi system introduces a time dimension to the original CSI tensor, which is a two-dimensional CSI tensor. The positioning results in the laboratory scene are shown in the following Figure 8:

In terms of localization results, MHSA-EC achieves the best localization results due to the introduction of effective CSI. The performance of 1dCNN-CSI and Confi are almost the same. The confidence of 1dCNN-CSI is higher in the range of 0–2 m, but the performance of Confi is better in the range of 8.2–10 m. Although the MHSA network is weaker than 1dCNN-CSI in the range of 3.8–4 m, the overall positioning error is better than the other two mainstream model structures. When the probability reaches 1.0, the positioning error of MHSA-EC is 7.23 m and MHSA is 8.12 m, while Confi is 9.36 m and 1dCNN-CSI is 9.53 m.

**Figure 9:**
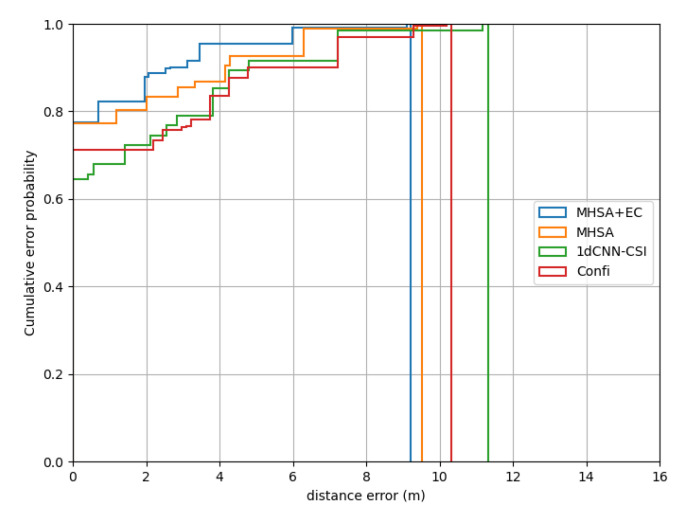
Performance on the comprehensive office.

The result of the comprehensive office scene is shown in the following Figure 9.

As shown in Figure 9, the localization performance of multiple localization methods in the comprehensive office scene is weaker than that in the laboratory scene. Overall, MHSA-based localization methods outperform Confi and 1dCNN-CSI. In the interval of 0–2 m, MHSA-EC is slightly better than the MHSA method, and in this long-distance error interval of (2 m, 6 m), the MHSA-EC network maintains higher confidence than other positioning systems. It can be shown that the introduction of effective CSI has a certain inhibitory effect on the mismatch of long-distance points. The confidence of Confi and the final positioning error within 0–1.5 m are higher than those of the 1dCNN-CSI system, and the performance of the two is close in other error intervals. When the probability reaches 1.0, the positioning error of MHSA-EC is 9.23 m and MHSA is 9.52 m, while Confi is 10.36 m and 1dCNN-CSI is 11.23 m.

In addition, we calculated the average positioning error and positioning standard deviation of the positioning system in different scenes to show the accuracy and stability of the positioning. The results are shown in Table 4. The MHSA-EC has the best accuracy and positioning stability. Compared with the Confi system, the accuracy is improved by 37%, and the stability is improved by 28% in the laboratory. In the comprehensive office, the accuracy is improved by 40%, and the stability is improved by 35%.

Experiments show that the MHSA-EC method outperforms other existing methods in localization accuracy. The introduction of the MHSA layer and effective CSI is of great help to the localization effect.

## 5. Summary

The ability to extract CSI amplitude features has a great impact on CSI-based positioning systems. This paper analyzes the difficulty of aggregating long-distance subcarriers in traditional convolutional networks and the problems of long-distance point mismatches. Based on these questions, this paper proposed an indoor localization algorithm fusing the multi-head self-attention mechanism and effective CSI. The algorithm architecture consists of a feature extraction module, a statistical module, a fusion, and a location decision module.

First of all, the multi-head self-attention layer is used in feature extraction to achieve a better aggregation effect for features with farther distances. The purpose of this is to better mine the location information contained in the multi-path effect.

At the same time, effective CSI is introduced to provide distance constraint information for the location decision module. Finally, the two kinds of information are integrated through the fusion module, and the final position coordinates are output by the decision module. MHSA-EC ensures the stability and accuracy of positioning. The average positioning error in the comprehensive office is 0.71 m, and the average positioning error in the laboratory is 0.64 m.

However, this paper only explores the CSI amplitude and does not introduce other signals. Compared with a single signal source, multi-source signals have more abundant location features and can provide complementary information to each other to ensure the accuracy and stability of the positioning algorithm. Therefore, other signal features can be fused with the CSI features extracted by the MHSA-EC algorithm in the future to improve the accuracy and stability of the positioning algorithm.

## Figures and Tables

**Table 1:** Parameters in the experiment.

Parameter Name	Parameter Value
batch size	16
learning rate	0.0005
nhead	5
loss function	Adam
initializer	Glorot initializer

**Table 2:** Multi-Head Self-Attention Layers Experiment.

MHSA Layers	MAE (m)	STD (m)
1	1.49	1.63
2	1.15	1.37
3	0.71	0.83
4	0.72	0.92

**Table 3:** Fusion method experiment.

Fusion Method	MAE (m)	Parameters (m)
sum	0.71	17
concat	0.82	19

**Table 4:** Comparison of localization effect with other methods.

Method	Lab.mae (m)	Lab.std (m)	Com.mae (m)	Com.std (m)
1dCNN-CSI	0.98	1.21	1.28	1.45
Confi	1.02	1.18	1.19	1.28
MHSA	0.83	0.96	0.91	1.08
MHSA-EC	0.64	0.85	0.71	0.83
